# Factors associated with breastfeeding intent among mothers of newborn babies in Da Nang, Viet Nam

**DOI:** 10.1186/s13006-017-0144-7

**Published:** 2018-01-11

**Authors:** Phuong Thi Kim Nguyen, Hoang Thi Tran, Thuy Thi Thanh Thai, Kirsty Foster, Christine L. Roberts, Ben J. Marais

**Affiliations:** 1 Discipline of Paediatrics and Adolescent Medicine, The Children’s Hospital at Westmead, The University of Sydney, Sydney, Australia; 2 Da Nang Hospital for Women and Children, Da Nang, Viet Nam; 3 Da Nang University of Medical Technology and Pharmacy, Da Nang, Viet Nam; 4 Office for Global Health, Sydney Medical School, The University of Sydney, Sydney, Australia; 5 Medical Education, Northern Clinical School, Sydney, Australia; 6 Clinical and Population Perinatal Health Research, Kolling Institute, Northern Sydney, Local Health District, Sydney, Australia

**Keywords:** Essential newborn care package, Breastfeeding, Viet Nam, Breastfeed

## Abstract

**Background:**

Breastfeeding is recognized as the single most cost-effective intervention to reduce child morbidity and mortality. However, few studies have explored perceived barriers to breastfeeding and factors associated with breastfeeding intent among mothers of newborn babies in Viet Nam. We conducted a study to assess breastfeeding initiation rates, intent to breastfeed exclusively for 6 months or more and perceived barriers to breastfeed among mothers of newborn babies in Da Nang, Viet Nam.

**Methods:**

We conducted a cross-sectional questionnaire survey of mothers in the postnatal wards of Da Nang Hospital for Women and Children in central Viet Nam from 10 February 2017 to 24 February 2017, following implementation of the World Health Organization (WHO) Essential Newborn Care (ENC) package.

**Results:**

Of 286 mothers surveyed, 259 (90.6%) initiated breastfeeding; 203/258 (78.7%) within 1 hour (h) of birth. Most (207, 72.4%) mothers indicated intent to breastfeed exclusively for 6 months or more, but this was lower among mothers of preterm babies (82.2% versus 20.0%, *p* < 0.001) and those without post-secondary school education (74.8% versus 55.6%, *p* = 0.02). Amongst mothers struggling to establish breastfeeding, 18/27 (66.7%) had a Cesarean section. Planned non-exclusive breastfeeding was mostly (39, 60.9%) motivated by mothers’ concern that their milk supply would be insufficient for their baby’s growth requirements. Most mothers had good knowledge about the benefits of breastfeeding and indicated strong decision autonomy.

**Conclusions:**

We documented high rates of early breastfeeding establishment and intent to breastfeed exclusively for 6 months or more. This probably reflects high levels of maternal education and successful implementation of the WHO ENC package. Mothers of premature babies may benefit from additional support.

**Electronic supplementary material:**

The online version of this article (10.1186/s13006-017-0144-7) contains supplementary material, which is available to authorized users.

## Background

Breastfeeding is recognized as the single most cost-effective intervention to reduce child morbidity and mortality [1, 2]. An estimated 13% of all under-5 mortality can be prevented by universal exclusive breastfeeding during the first 6 months of life [3, 4]. Studies conducted in the World Health Organization (WHO) Western Pacific region support the benefits attributed to exclusive breastfeeding [5, 6]. However, global estimates indicate that only 36% of infants born in 2015 were exclusively breastfed during the first 6 months of life [7], with decreasing breastfeeding rates observed in some of the most populous countries like China, India, Indonesia, Pakistan, Nigeria, Bangladesh, Mexico, the Philippines and Viet Nam [1, 8, 9]. Known barriers to breastfeeding uptake include socio-cultural factors, poor health education and inaccurate information about the “benefits” of formula milk [10–12]. A study in five Asian countries (Viet Nam, Timor-Leste, The Philippines, Indonesia, Cambodia) identified maternal employment, increased maternal age and low maternal education as risk factors for formula or non-exclusive breastfeeding [13]. In rural Viet Nam, mothers who worked away from home were most likely not to breastfeed exclusively at an early age [14].

In 2014, the WHO Western Pacific region and the United Nations Children’s Fund (UNICEF) formulated an action plan to reduce infant mortality by increasing the prevalence of breastfeeding [15, 16], using the WHO Essential Newborn Care (ENC) package. The ENC package includes four steps; 1) immediate drying of the newborn baby, 2) establishing skin-to-skin contact with the mother as soon as possible after delivery, 3) delayed cord clamping and 4) breastfeeding within 1 h of delivery-birth [17]. Several breastfeeding promotion initiatives have been launched in Viet Nam including health education programs, the establishment of “baby-friendly” hospitals [18], and the implementation of the ENC package [19].

The Da Nang Hospital for Women and Children in central Viet Nam achieved “baby-friendly” status, after implementing the ENC package. Before introducing the package, only 40% of newborns breastfed within 1 h of life, and similar to other settings in Viet Nam less than a third (27%) of mothers breastfed exclusively during the neonatal period [20]. Early breastfeeding initiation rates, factors related to longer term breastfeeding intent and perceived barriers to exclusive breastfeeding have not been formally assessed in this setting after introduction of the ENC package.

## Methods

We conducted a cross-sectional survey of mothers in the postnatal wards, within the first few days after delivery, to assess breastfeeding initiation rates, intent to breastfeed exclusively for 6 months or more and perceived barriers to exclusive breastfeeding. The study was performed over a 2-week period (10 February 2017 to 24 February 2017). All participating mothers provided written informed consent.

### Study setting

The Da Nang Hospital for Women and Children is a regional hospital in central Viet Nam with 1675 inpatient beds. It is the largest provider of obstetric services in Da Nang city with around 14,000 deliveries each year. It also serves as the regional referral hospital for women and children. Pregnant women may come directly from home or be transferred from surrounding district and provincial hospitals. The Da Nang Hospital for Women and Children has fully implemented the WHO ENC package since November 2014 [17].

### Data collection and analysis

A detailed case report form was used to guide data collection, including basic demographic and clinical information as well as 10 questions constructed as yes/no and multiple-choice options (see Additional file 1 for the questionnaire). All questions were critically assessed and refined to standardize data collection and limit possible misinterpretation. A group of five Vietnamese mothers assisted questionnaire development to ensure that questions were correctly interpreted in the local cultural context. All definitions were clarified in advance with exclusive breastfeeding defined as “breastmilk only”, excluding any infant formula, nutritional supplements or water, for a minimum period of 6 months [4]. To optimize the accuracy of the translated questionnaire, back translation into English was performed by a second independent translator and verified by the lead investigator. Final refinements were made after pilot testing involving more than 30 mothers. The questionnaire was completed as a structured interview performed in the postnatal ward within 48 h of delivery-birth.

All mothers present in the postnatal wards when the researchers made their daily visits were recruited, if they consented to participate in the study. Data were collected by the lead investigator (PTKN) and a trained assistant (TTTT). Study personnel were not involved in the mothers’ clinical care. There were no exclusion criteria and mothers were recruited irrespective of their date or mode of delivery. We recorded basic demographics characteristics (age, education level, address, type of delivery and reason for refusal) from all mothers who were unavailable for recruitment (refused participation or were absent when the study team visited) from the ward admission book. De-identified data were entered into an EpiData database [21] and both descriptive and comparative analyses performed using SPSS (Version 24 IBM, NY, USA). For comparative analyses we classified mothers into 2 groups, 1) those who indicated an intent to breastfeed exclusively for 6 months or more (exclusive BF) and 2) those who had not initiated breastfeeding at the time of interview or indicated that they intended to mix feed or breastfeed for less than 6 months (non-exclusive BF).

## Results

**Fig. 1:**
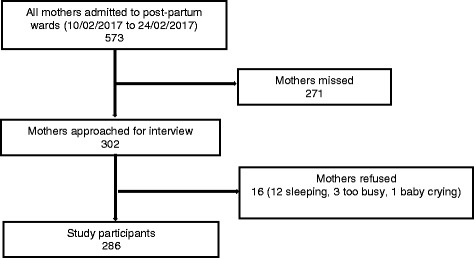
Flow diagram of study recruitment

In total, 573 mothers were admitted to the postnatal wards during the 2-week study period, of whom 302 were aproached and 286/302 (94.7%) completed the questionnaire (Fig. 1). Only 16 (5.3%) mothers declined study participation, mostly because they were too tired to be interviewed. The average age, occupation, mode of delivery and rural or urban origin were not statistically different between study participants and those who were not enrolled in the study (Additional file 2). All study participants were from the majority Kinh ethnic group.

**Fig. 2:**
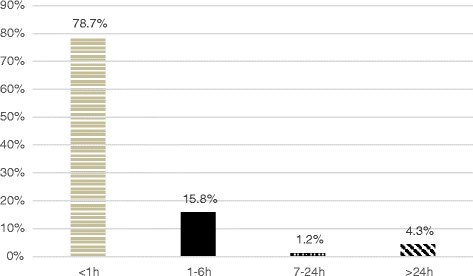
Time from delivery to first breastfeeding attempt

At the time of interview, 259/286 (90.6%) mothers had initiated breastfeeding and 207 (72.4%) indicated that they intended to breastfeed exclusively for 6 months or more. Nearly 80% of mothers (203/258, 78.7%) initiated breastfeeding within the first hour after delivery-birth (Fig. 2), including the majority (169/207; 81.6%) of mothers who intended to breastfeed exclusively for 6 months or more. Among 52 mothers who intended to breastfeed exclusively for less than 6 months, 12 (4.6%) indicated a planned duration of 4–5 months, 3 (1.2%) 2–3 months and 27 (10.4%) indicated a preference for non-exclusive breastfeeding. A small percentage (10, 3.9%) of mothers were unsure how long they planned to breastfeed; most (9/10; 90.0%) were first-time mothers.

Table 1 compares the demographic characteristics of mothers who intended to breastfeed exclusively for 6 months or more and those who did not. There were no significant differences in the mothers’s age, area of residence, employment status, parity, the infants ‘birth weight’ number of babies or mode of delivery. Mothers were generally well educated, but those without post-secondary school education were slightly less likely to express an intent to breastfeed exclusively for 6 months or more. The biggest difference was observed in mothers of preterm babies (< 37 weeks gestation), who were less likely to consider exclusive breastfeeding for 6 months than mothers of full term infants. Among 27 mothers struggling to establish breastfeeding, most (18, 66.7%) had a Cesarean section and indicated that they had tried unsuccessfully (16/18, 88.9%) or were too tired to breastfeed (2/18, 11.1%).

**Table 1:** Characteristics of mothers who planned to exclusively breastfeed for 6 months compared to those who did not

Characteristics	Exclusive BF^a^ *n* = 207 n (row %)	Non-exclusive BF^b^ *n* = 79 n (row %)	Total *n* = 286 n (column %)	PR (95% CI)	*p*-value
Age (years)					
≥ 35	34 (69.4)	15 (30.6)	49 (17.1)	1	
< 35	173 (73.0)	64 (27.0)	237 (82.9)	1.1 (0.9–1.3)	0.6
Area of residence					
Rural	94 (72.3)	36 (27.7)	130 (45.5)	1	
Urban	113 (72.4)	43 (27.6)	156 (54.5)	1.0 (0.9–1.2)	1.0
Education					
After secondary school	187 (74.8)	63 (25.2)	250 (87.4)	***1***	
Up to secondary school	20 (55.6)	16 (44.4)	36 (12.6)	***0.7 (0.6–1.0)***	***0.02***
Employment status					
Not employed	42 (77.8)	12 (22.2)	54 (18.9)	1	
Current employment	165 (71.1)	67 (28.9)	232 (81.1)	0.9 (0.8–1.1)	0.3
Parity					
More than 1 child	97 (75.8)	31 (24.2)	128 (44.8)	1	
First child	110 (69.6)	48 (30.4)	158 (55.2)	0.9 (0.8–1.1)	0.2
Previous BF^c^					
No	7 (53.8)	6 (46.2)	13 (10.3)	1	
Yes	88 (77.9)	25 (22.1)	113 (89.7)	1.4 (0.9–2.4)	0.06
Gestational age					
≥ 37 weeks	198 (82.2)	43 (17.8)	241 (84.3)	***1***	
< 37 weeks	9 (20.0)	36 (80.0)	45 (15.7)	***0.2 (0.1–0.4)***	***< 0.001***
Birth weight					
≥ 2500 g	199 (72.4)	76 (27.6)	275 (96.2)	1	
< 2500 g	8 (72.7)	3 (27.3)	11 (3.8)	1.0 (0.7–1.5)	1.0
Number of babies					
Singleton	204 (72.1)	79 (27.9)	283 (99.0)	1	
Twins	3 (100.0)	0	3 (1.0)	0.7 (0.6–0.8)	0.6
Mode of delivery					
Vaginal	86 (69.4)	38 (30.6)	124 (43.4)	1	
Cesarean section	121 (74.7)	41 (25.3)	162 (56.6)	1.1 (0.9–1.2)	0.3

*BF* breastfeeding, *PR* prevalence ratio, *CI* confidence interval

^a^Breastfeeding established and intent to breastfeed exclusively for 6 months or more

^b^All other mothers including those in whom breastfeeding was not established at the time of interview

^c^Missing data for two mothers

Table 2 summarises the factors that influenced mothers’ breastfeeding decision. Belief that “breast is best” was the most common reason, followed by improved mother-infant bonding. Cost considerations were not a major factor. The majority of mothers (208; 72.7%) indicated decision autonomy, stating that they got advice from health professionals, their husband, mothers or nannies, but ultimately made up their own minds. Informally, mothers indicated that they also accessed information via the internet and television.

**Table 2:** Factors that influenced mothers’ breastfeeding decision

Factors		Most important	Second most important^*^
		*n* (%)	*n* (%)
In mothers who were breastfeeding (*n* = 259)			
Reasons for breastfeeding my child	I believe breastmilk is the best	203 (78.4)	20 (13.6)
	It improves mother-infant bonding	25 (9.7)	73 (49.7)
	It is hygienic	14 (5.4)	32 (21.8)
	It is convenient	9 (3.5)	15 (10.2)
	It is economical	6 (2.3)	7 (4.8)
	Other^a^	2 (0.8)	NA
Main people who influenced my decision^**^	I made the decision myself	161 (62.4)	9 (10.3)
	Health professional	42 (16.3)	21 (24.1)
	Husband	36 (14.0)	29 (33.3)
	Mother(s)	13 (5.0)	27 (31.0)
	Nanny	6 (2.3)	1 (1.1)
In mothers who were NOT breastfeeding (*n* = 27)			
Reasons for not breastfeeding my child	I have tried unsuccessfully	22 (81.5)	0
	I am too tired to breastfeed	2 (7.4)	3 (75.0)
	Other^b^	3 (11.1)	1 (25.0)
Main people who influenced my decision	I made the decision myself	25 (92.6)	0
	Mother(s)	2 (7.4)	0

*NA* not applicable

^*^This does not necessarily add up to N, since many mothers failed to indicate the second most important factor; ^**^Data was missing for 1

^a^Breastmilk protects my child from diseases; My child refused formula milk

^b^My child was unable to latch successfully (× 2); I have not had milk since giving birth, but will try again

Among mothers who did not establish breasteeding at the time of interview, most (22/27; 81.5%) indicated that they had tried unsuccessfully, listing reasons such as the baby crying unconsolably and not latching properly, or not having sufficient milk. Table 3 demonstrates that mothers‘had good general knowledge about breastfeeding benefits. Table 4 reflects mothers’ reasons for considering non-exclusive breastfeeding during the first 6 months and planned breastmilk supplements. The main reason for considering non-exclusive breastfeeding was concern that breastmilk alone may be insufficient (39/64, 60.9%). Infant formula (62/67, 92.5%) was the main breastmilk substitute or supplement considered.

**Table 3:** Mothers’ knowledge of breastfeeding risks and benefits

Question (*n* = 286)	Answer		
	Yes	No	Unsure
	*n* (%)	*n* (%)	*n* (%)
Does breastmilk contain all the nutrients an infant needs during the first 6 months?	**279 (97.6)**	3 (1.0)	4 (1.4)
Does breastfeeding increase the infant’s risk of diarrhoea?	14 (4.9)	**262 (91.6)**	10 (3.5)
Does breastfeeding strengthen the bonding between a mother and her baby?	**281 (98.3)**	4 (1.4)	1 (0.3)

Correct answers in bold

**Table 4:** Mothers’ reasons for considering non-exclusive breastfeeding during the first 6 months and planned breastmilk supplements

Reasons for considering non-excusive breastfeeding during the first 6 months (*n* = 64)^*^	First choice	Second choice^**^
	*n* (%)	*n* (%)
I think my breastmilk is unlikely to be enough and my child will be hungry	39 (60.9)	5 (50.0)
I have to restart work and cannot continue exclusive breastfeeding	11 (17.2)	2 (20.0)
I think my breastmilk does not provide all the necessary vitamins & supplements	5 (7.8)	3 (30.0)
Other^a^	9 (14.1)	NA
Planned breastmilk supplements (*n* = 67)^*^		
Formula milk	62 (91.1)	4 (20.0)
Water	4 (5.9)	5 (25.0)
Vitamins & supplements	0	1 (5.0)
Herbal tea	0	0
Solids	1 (1.5)	10 (50.0)

^*^Only answered by mothers who indicated that they did not intend to breastfeed exclusively for 6 months; not all mothers answered all questions

^**^This does not add up to *n*, since many mothers did not indicate a second choice

^a^I have not had milk since giving birth

## Discussion

We documented a high prevalence of early breastfeeding initiation following successful implementation of the WHO Essential Newborn Care (ENC) package. Previous studies in Viet Nam showed a lower rate (40–60%) of breastfeeding initiation within 1 h of birth [8]. Early initiation of breastfeeding is associated with significantly reduced neonatal mortality [22], and breastfeeding in general with reduced under-5 mortality [3], mediated by less acute respiratory infections, diarrhoea and malnutrition [2, 23]. Delayed breastfeeding initiation often leads to non-exclusive breastfeeding [24, 25]. In Viet Nam, the use of infant formula during the first 3 days of life has been associated with early breastfeeding cessation even in mothers who were able to establish breastfeeding [26]. Nearly three quarters of all the mothers’ interviewed intended to provide exclusive breastfeeding for 6 months or more, which is much higher than previous (2007–2014) national estimates of 17% [7]. While stated intent is not necessarily representative of actual practice, it should provide a fairly reliable proxy.

We were interested in factors that influenced the mothers‘breastfeeding decision, since the preferences of her mother and husband carries great weight in Confucian cultures [11, 18]. Most mothers consulted with health professtionals and close family members, but displayed strong decision autonomy. Mothers chose breastfeeding as they believed “breast is best” and to improve mother-infant bonding. Mothers’ concern that their milk may not supply everything that their baby needs has been noted before [27], and may be influenced by the marketing tactics used by companies selling “enhanced” infant milk formulas. It is important to clearly articulate and communicate the benefits of exclusive breastfeeding to mothers [28], emphasizing the fact that it provides sufficient quantity and nutritional quality for a growing baby. Reliable health information should also be made available on trusted websites, given that the internet is an important source of health information for educated mothers [29].

The rate of Cesarean section delivery was high (more than 50% mothers had this mode of delivery) compared to other countries in the region [7]. Although Da Nang hospital for Women and Children is a regional referral hospital with a more complex patient mix, high Cesarean section birth in Viet Nam reflect local clinical guidance and cultural expectations. Mothers who underwent a Cesarean section were less likely to initiate breastfeeding than those who delivered vaginally, which may be related to concerns that antibiotics can be transferred to their babies via breastmilk, while post-operative pain, fatigue, and limited mobility also reduce the impetus of Cesarean section mothers to breastfeed [18, 30, 31].

In this study preterm birth delivery was the only major factor associated with a reduced likelihood of intent to breastfeed exclusively for 6 months, although the number of pre-term infants were relatively small. It is especially important for preterm babies to be breastfed, given the multiple beneficial effects of breastfeeding on infectious disease risk, growth and neurocognitive development [32]. The Da Nang Hospital for Women and Children introduced kangaroo mother care to support preterm babies [33], but the rate of breastfeeding and intent to exclusively breastfeed for 6 months or more was found to be significantly lower among preterm (< 37 weeks) compared to full-term infants. We cannot comment on babies with pronounced prematurity, but our findings suggest that extra effort should be made to encourage exclusive breastfeeding of preterm babies who do not require special care, since mothers may perceive them to have increased requirements for breastmilk supplementation.

In terms of planned breastmilk supplements, mothers mostly indicated the use of infant formula, water and solids, which is similar to previous findings [34]. Few mothers considered vitamin and micronutrient supplementation. Not a single mother indicated the use of herbal tea, which has been reported as a common supplement among ethnic minority groups in Viet Nam [34, 35].

Our study was limited by the fact that we were unable to interview all mothers who delivered during the study period, due to the limited number of interviewers available. However, we tried to limit potential selection bias and did not find any statistically significant differences between mothers who completed the questionnaire and those not included in the study (Additional file 2). Interviews were completed within the first few days after birth and the hospital environment may have influenced mothers to provide the answers that they thought health care providers wanted. However, mothers indicated strong decision autonomy, which argues against potential “social desirability bias”. Our study did not include any women from minority ethnic groups and therefore the study findings cannot be extrapolated to these populations, women from minority ethnic groups may have different barriers to exclusive breastfeeding [34]. We only documented breastfeeding initiation and intent and the lack of data on actual breastfeeding duration is a major limitation that has to be acknowledged.

## Conclusions

We documented high rates of breastfeeding initiation and intent to breastfeed exclusively for 6 months or more. Mothers were generally well educated with high levels of decision autonomy. Mothers of preterm babies may benefit from enhanced support to encourage exclusive breastfeeding.

## Additional files

Additional file 1: Questionnaire. (DOCX 34 kb)

Additional file 2: Table S1. Comparison of demographic characteristics s of mothers who did and did not complete the postpartum questionnaire during the study period. (DOCX 13 kb)
