# Supplementary material for: Factors associated with breastfeeding intent among mothers of newborn babies in Da Nang, Viet Nam
Source: Int Breastfeed J. 2018 Jan 11;13:2. doi: 10.1186/s13006-017-0144-7 (PMC5765663; doi:10.1186/s13006-017-0144-7)
Supplement: Supplementary file 1 — Questionnaire. (DOCX 34 kb) [file 13006_2017_144_MOESM1_ESM.docx]

Number:………….. ID Number:………….. Interviewer:……………………… Date:……………

**Factors associated with breastfeeding practice among mothers of newborn babies**

**in Da Nang, Viet Nam**

**Child** Age: …… day(s) Sex: Male☐ Female☐ Gestation age: …….w(s) P:...……gram

Singleton: Y☐ N☐ Skin to skin contact: Y☐ N☐ HBV: Given ☐ Not given ☐

Mode of delivery: C-section☐ Vaginal ☐ PARA: ☐☐☐☐

**Mother** Age: ……years Place: Urban☐ Rural☐ RVD status: Pos☐ Neg☐ Not tested ☐

**Questions**

Work: Formally employed ☐ Informally employed ☐ Unemployed ☐

Education: None ☐ Primary ☐ Secondary ☐ High school ☐ College/ University ☐

*1. Are you currently breastfeeding?* Y☐🡪 move to question 3 N☐

*2. If no - what are the reasons for not breastfeeding? (check up to 2 replies for each column - ranked 1,2)*

I don’t want to breastfeed ☐ Husband’s advice ☐

I believe formula milk is the best ☐ Mothers’advice ☐

I have tried unsuccessfully ☐ Health professional’s advice ☐

I am too tired to breastfeed ☐ Nanny’s advice ☐

I am scared of cultural breast massage ☐ Myself decision ☐

Other (specify or elaborate)……………………………………………………………………………………………………………

🡪 move to question 9

*3. If yes - what are the reasons that you are breastfeeding? (check up to 2 replies for each column - ranked 1,2)*

It is convenient to breastfeed ☐ Husband’s advice ☐

It enhances mother-infant bonding ☐ Mothers’advice ☐

I believe breastmilk is the best ☐ Health professional’s advice ☐

It is economical to breastfeed ☐ Nanny’s advice ☐

Breastfeeding is hygienic ☐ Myself decision ☐

Other (specify or elaborate)……………………………………………………………………………………………………………

*4. How long after delivery did you first breastfeed?*

Within 1 hour☐ 2-6 hours☐ 7-24 hours☐ >24 hours☐

*5. Do you intend to breastfeed exclusively?*  Y☐ N☐🡪 move to question 7

*6. How long do you intend to breastfeed exclusively?*

<2 months☐ 2-3 months☐ 4-5 months☐ 6 months or more ☐ Not sure ☐

🡪 move to question 9

*7. If not breastfeeding exclusively, which of the following would you consider introducing during the first 6 months (check up to 3 – ranked 1,2,3)*

Water ☐ Vitamins & supplements ☐ Herbal tea ☐ Formula milk ☐ Solids (rice porridge etc.)☐

*8. Why would you consider non-exclusive breastfeeding in the first 6 months? (check all that apply – ranked 1,2,3)*

I think my breastmilk does not provide all the necessary vitamins & supplements ☐

I think my breastmilk is unlikely to be enough and my child will be hungry ☐

I have to re-start work and cannot continue exclusive breastfeeding ☐

Other (specify or elaborate)……………………………………………………………………………………………………………

*9. If you had previous children did you breastfeed them exclusively?*

Child 1: Y☐ N☐ If yes, how long did you breastfeed? ...........months

Who influenced your breastfeeding decision with this pregnancy: *………………………………………………………………*

Child 2: Y☐ N☐ If yes, how long did you breastfeed? ...........months

Who influenced your breastfeeding decision with this pregnancy: *………………………………………………………………*

10. *Please answer the following questions:*

Does breastmilk contain all the nutrients an infant needs in the first 6 months? Y☐ N☐ Unsure☐

Does breastfeeding increase the infant’s risk of diarrhea? Y☐ N☐ Unsure☐

Does breastfeeding protect an infant against HIV infection? Y☐ N☐ Unsure☐

Does breastfeeding protect an infant against lung infections? Y☐ N☐ Unsure☐

Does breastfeeding strengthen the bonding between a mother and her baby? Y☐ N☐ Unsure☐

Any additional comments?......................................................................................................................................................

Thank you so much!
