# Supplementary material for: Factors associated with breastfeeding intent among mothers of newborn babies in Da Nang, Viet Nam
Source: Int Breastfeed J. 2018 Jan 11;13:2. doi: 10.1186/s13006-017-0144-7 (PMC5765663; doi:10.1186/s13006-017-0144-7)
Supplement: Supplementary file 2 — Comparison of demographic characteristics s of mothers who did and did not complete the postpartum questionnaire during the study period. (DOCX 13 kb) [file 13006_2017_144_MOESM2_ESM.docx]

**Table S1. Comparison of demographic characteristics s of mothers who did and did not complete the postpartum questionnaire during the study period**

| **Demographics** | | **Mothers not included in study**  **n (%)** | **Study participants**  **n (%)** | **Total** | **p** |
| --- | --- | --- | --- | --- | --- |
| **Age (years)** | 15-19  20-24  25-29  30-34  35-39  >40 | 5 (71.4)  39 (43.8)  133 (52.8)  76 (53.5)  29 (46.8)  9 (42.9) | 2 (28.6)  50 (56.2)  119 (47.2)  66 (46.5)  33 (53.2)  12 (57.1) | 7 (1.2)  89 (15.5)  252 (44.0)  142 (24.8)  62 (10.8)  21 (3.7) | >0.05 |
| **Place of residence ^a^** | Urban  Rural | 159 (50.5)  128 (49.6) | 156 (49.5)  130 (50.4) | 315 (55.0)  258 (45.0) | >0.05 |
| **Occupation ^b^** | Employed  Self-employed  Un-employed | 155 (49.5)  75 (50.3)  57 (51.4) | 158 (50.5)  74 (49.7)  54 (48.6) | 313 (54.6)  149 (26.0)  111 (19.4) | >0.05 |
| **Mode of delivery** | Caesarean section  Vaginal delivery | 165 (50.4)  122 (49.6) | 162 (49.6)  124 (50.4) | 327 (57.1)  246 (42.9) | >0.05 |
| **Total** | | 287 (50.0) | 286 (50.0) | 573 |  |

^a^ Place of residence - Urban: administrative units (communes) that are part of recognized cities or large towns; rural: all other administrative units (communes);

^b^ Occupation - Employed: have a paying job and will return to this job after maternity leave; self-employed: generate own income; unemployed: no income generation
